# Silent Signals in the Snow: Tracking the Spatio‐Temporal Territorial Marking Behavior of Snow Leopards (*Panthera uncia*) in the Mountainous Region of Baltistan, Pakistan

**DOI:** 10.1002/ece3.70518

**Published:** 2024-12-11

**Authors:** Muhammad Zaman, Yi Chen, Rodney Jackson, Shafqat Hussain

**Affiliations:** ^1^ Institude of Evolutionary Ecology and Conservation Biology Central South University of Forestry and Technology Changsha Hunan China; ^2^ Baltistan Wildlife Conservation and Development Organization (Reg) Apixoq Abbas Town Skardu Gilgit Baltistan Pakistan; ^3^ Snow Leopard Conservancy Sonoma California USA; ^4^ Trinity College Hartford Connecticut USA

**Keywords:** camera trap, communication behaviors, snow cover, snow leopard, temporal overlap

## Abstract

Mammals, being social creatures communicate through a variety of signal cues, thus it is vital to understand how wild carnivores create and maintain connections with their neighbors for their survival. However, observing elusive species in their natural habitats poses significant challenges leading to scarcities of data. In this study, we aimed to provide a detailed long‐term observation of snow leopards in the northern region of Pakistan, hence we utilized data from 136 camera traps between 2018 and 2023 in order to investigate the territorial marking behavior of snow leopards in Baltistan. We documented 813 sightings of snow leopards with 103 videos showing territorial marking behavior from 40 sightings during snow presence and 63 on snow‐free days. Nine unique communication behaviors were identified during the presence or absence of snow cover. We observed that snow leopard marking behavior occurred more often at night in the absence of snow and less often during the day in the presence of snow. The marking activities were higher during the crepuscular period in the presence of snow and showed a preference for marking activities in open areas over mixed herbaceous and shrub habitats. Olfaction and scraping were observed more frequently in open areas while fecal deposition was found in herbaceous and shrub habitats. Scraping and urine spraying were associated with cliffs, rocky outcrops and boulders in open areas. In conclusion, our findings discovered new insights into the marking patterns of snow leopards during both day and night, having taken into consideration the influence of snow conditions. Moreover, the identified marking locations hold significant potential as powerful assets for wildlife preservation initiatives.

## Graphical abstract

Mammals exhibit complex social behaviors by utilizing various forms of communication such as visual cues, acoustic signals and olfactory sensations. The communication patterns within mammalian species play a crucial role in their behavioral ecology, ultimately influencing their overall health and survival. However, studying communication behaviors and observing elusive species in their natural habitats present significant challenges.

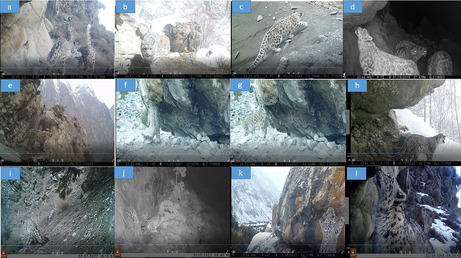

## 1 Introduction

Mammals are social creatures that communicate through various signal cues, including visual, acoustic and olfactory signals (Mináriková et al. 2023). These cues help them establish and maintain spatio‐temporal connections with their neighbors similar to railway signals guiding the movement of trains (Newsome et al. 2017). The decay of olfactory signals due to factors like precipitation, snowstorms, ultraviolet radiation and bacterial decomposition varies across seasons (Parsons et al. 2018), with signal cues degrading faster during wet periods compared to dry periods (Pal 2003). This heightened decay of signal cues during certain periods leads to increased territorial defense costs for mammals (Parsons et al. 2018; Rosenbaum et al. 2023). Felids use a variety of scent‐marking behaviors to communicate such as urine spraying, scratching, defecation, claw marking and rubbing their bodies (Allen, Wallace, and Wilmers 2015; Allen et al. 2015, 2016), as well as territorial calling during the mating season (Richardson 1993). This communication method among felids is likely used to avoid unnecessary conflicts within and between species by marking their territory and sharing information to improve mating opportunities across different spaces and times (Allen, Wallace, and Wilmers 2015; Allen et al. 2015; Smith, Mcdougal, and Miquelle 1989). Temporal changes in the effectiveness of scent markings are also an important issue discussed in the theoretical framework of territoriality and spatial resource separation (Lewis and Moorcroft 2001). Scent‐based communication is widely considered the primary mode of interaction among felines. This phenomenon has been documented in various species of felids, such as tigers (*Panthera tigris*) (Smith, Mcdougal, and Miquelle 1989), servals (*Leptailurus serval*) (Geertsema 1984), domestic cats (*Felis catus*) (Feldman 1994), black‐footed cats (*Felis nigripes*) (Sliwa et al. 2018), pumas (*Puma concolor*) (Allen, Wittmer, and Wilmers 2014), bobcat cats (*Lynx rufus*) (Allen, Wallace, and Wilmers 2015; Allen et al. 2015), sunda clouded leopards (*Neofelis diardi*) (Maximilian L. Allen et al. 2016), persian leopards (*Panthera pardus saxicolor*) (Ghoddousi et al. 2008; Hunter, McCarthy, and McCarthy 2016), snow leopards (Macri and Patterson‐Kane 2011) and various other small felids (Mellen 1993).

In a mountainous landscape, exploring the timing and locations of scent marking among wild animals presents significant methodological challenges (Ahlborn and Jackson 1986; Bidder et al. 2020). Studying the communication behaviors of elusive species in their natural environments, particularly carnivores inhabiting remote and difficult‐to‐access areas, has historically been problematic and lacking in information (Allen et al. 2016). Currently, there is a lack of basic information on communication behavior with data entirely lacking for 23% of all felid species (Allen et al. 2016) and a notable gap in understanding scent marking behavior during both day and night in the wild (Rafiq et al. 2020). Studying solitary carnivores through direct observation has provided valuable insights into their spatio‐temporal distribution and the environmental context for scent marking (Allen, Bekoff, and Crabtree 1999). However, this method may be influenced by observer bias (Tuyttens et al. 2014), limited to specific areas of observation (Jordan et al. 2013), and less effective or time consuming for secretive (or rarely sighted) species with large home ranges (Ahlborn and Jackson 1986; Zaman et al. 2020). In order to gain a deeper understanding of how animals mark their territories over time and space, researchers have traditionally relied on tracking scent markings left behind in the snow (Rothman and Mech 1979). This approach may not provide accurate insights into identifying individual animals particularly in solitary species where scent marking behavior and strategies can vary depending on factors such as their social hierarchy status and mating or breeding time (Johansson et al. 2021; Sillero‐Zubiri and Macdonald 1998). The tracking of animals in snowy conditions presents challenges due to its unsuitability for warm climates or areas without snow (Bidder et al. 2020), and this limitation is particularly concerning for rare species that inhabit steep and rocky, high mountainous habitats (Rosenbaum et al. 2023). Research on animal communication has primarily focused on easily detectable visual and auditory signals neglecting the study of olfactory communication tools during different time periods (Bidder et al. 2020). This lack of attention to olfactory communication is especially problematic for felids as they are cryptic and may travel long distances across challenging terrain, are active at night or rely heavily on olfactory signals (Li et al. 2013; Rafiq et al. 2020). The communication through olfactory cues remains largely uncharted for nearly a quarter of felid species (Maximilian L. Allen et al. 2016). In fact, our understanding of olfactory behaviors in many species is mostly limited to observations in captive animals, while data gathered from wild populations remains notably scarce (Mellen 1993). This scarcity is primarily due to challenges in visually observing certain elusive species as noted by Ahlborn and Jackson (1986). Emerging innovative field techniques for analyzing scent‐marking behaviors have the potential to open up new avenues for ecological research leading to a deeper understanding and insight into cryptic animal behavior in the wild (Allen et al. 2016).

In disturbed environments influenced by pastoralism, the presence patterns and scent marking behaviors of carnivorous animals play a crucial role in influencing population levels across different time periods and geographical areas (Ahlborn and Jackson 1986; Sharma, Bhatnagar, and Mishra 2015). Despite ongoing efforts to investigate the behavior of wild snow leopards in mountainous regions, there remains a significant gap in our understanding of their presence patterns and marking behaviors over time (Ahlborn and Jackson 1986; Johansson et al. 2022). This knowledge gap encompasses both diurnal and nocturnal behaviors displayed by these elusive predators (Li et al. 2023; Noor et al. 2017). Moreover, there is a significant gap in our understanding of how snow leopards adjust their territorial markings throughout the day or under snowy conditions (Rokaya, Timsina, and Kindlmann 2022). These elusive felines also face challenges with fluctuating thermoregulation particularly in regions experiencing rapid climate change (Bilodeau, Gauthier, and Berteaux 2013). Previous research has shown that snow leopards exhibit social marking behavior as documented by Schaller (1977) in captivity as well as in observations of wild snow leopards by Wemmer and Scow (1977). Nevertheless, Ahlborn and Jackson (1986) noted that identifying the temporal marking patterns of snow leopards remains difficult particularly in the rugged terrain and steep landscapes that they occupy. Intriguingly, snow leopards possess the remarkable ability to modify their daily and seasonal activity patterns based on snow availability (Rosenbaum et al. 2023). When snow is plentiful during winter months, these majestic creatures adjust their behavior to effectively regulate body temperature, thus facilitating survival in this harsh environments (Farrington and Li 2024). On the contrary, during the summer months when snow amounts are limited, snow leopards adjust their behavior accordingly and show a preference for high‐altitude regions (Rosenbaum et al. 2023). Despite this ability to adapt to changing weather conditions being crucial for snow leopards, there is still a lack of research on how snow leopards deal with environmental challenges (Bilodeau, Gauthier, and Berteaux 2013; Johansson et al. 2022). Understanding how snow leopards utilize various habitats such as ridges, riverbeds and cliffs during the presence or absence of snow and according to day/night time for marking their territories is crucial for the behavioral study of this rare species (Aryal et al. 2014; Li et al. 2013).

In this study, we used non‐invasive camera trapping methods to monitor the marking of snow leopard territories over multiple years in four districts: Shigar, Gangche, Skardu, Rondo and Basho (Zaman, Jackson, and Hussain 2024; Zaman et al. 2024). The use of these techniques is essential for maintaining the balance of mountain ecosystems by tracking the spatial and temporal patterns of territorial marking behavior of snow leopards (Jackson et al. 2006; Sharief et al. 2022). These methods are particularly effective for gathering data on rare species that inhabit rugged and remote environments at various scales of time and space (Li et al. 2023; Liu et al. 2022). However, the monitoring and detectability of snow leopards through camera traps can be influenced by various factors including human activities such as changes in land use (Buzzard, Li, and Bleisch 2017), climatic factors (Johansson et al. 2022), grazing pressure (Li et al. 2023), poaching (Hacker et al. 2021; Li and Lu 2014), retaliatory killings due to livestock damage and the fur trade (Hussain 2003), as well as the reduction in prey populations (Li et al. 2021). These factors are significantly impacting the population of endangered snow leopards in the northern mountainous region of Pakistan (Zaman, Jackson, and Hussain 2024; Zaman et al. 2024). Consequently, snow leopards have been listed under appendix I of the Convention on International Trade in Endangered Species (CITES) (Hussain 2003).

Currently, there is a lack of accessible information on the spatio‐temporal territorial marking behavior of wild snow leopards in Pakistan. Thus, understanding the marking behaviors of snow leopards is crucial for developing effective monitoring methods as these indicators are commonly used to assess population size and spatial distribution (Ahlborn and Jackson 1986; Jackson et al. 2006). It is likely that changes in snow cover due to climate change (Bilodeau, Gauthier, and Berteaux 2013; Karimov, Kachel, and Hackländer 2018) and availability of habitat resources (Johansson et al. 2016) can impact the frequency of temporal activity and marking behaviors. Factors such as communication behavior (Ahlborn and Jackson 1986) and spatial disturbances are essential for the conservation of carnivore species (Johansson et al. 2021). The primary goal of this study is to present field‐based data gathered over several years in the human‐influenced environment of Baltistan. Additionally, we have successfully documented the first evidence of snow leopard spatio‐temporal activity and territorial marking in snowy conditions. Moreover, we examine the effects of snow cover on territorial marking behaviors (Mellen 1993) by considering the anticipated impacts of climate change on animal behavior patterns as mentioned by Johansson et al. (2022). This study focuses on three objectives: first, we explore the daily marking activity patterns of snow leopards including any potential differences in territorial marking between day and night in their habitats. Second, we investigate how snow cover during winter influences the daily marking activity patterns of snow leopards during day, night and twilight periods. Third, we will explore the impact of temperature changes on the preference or avoidance of peak marking activity by snow leopards. Specifically, we will examine how fluctuations in temperature may lead to a decrease in nighttime activity prompting a shift to daytime behavior in order to adapt to changing climatic conditions (Farrington and Li 2024; Li et al. 2016).

## 2 Materials and Methods

### 2.1 Study Area

**FIGURE 1:**
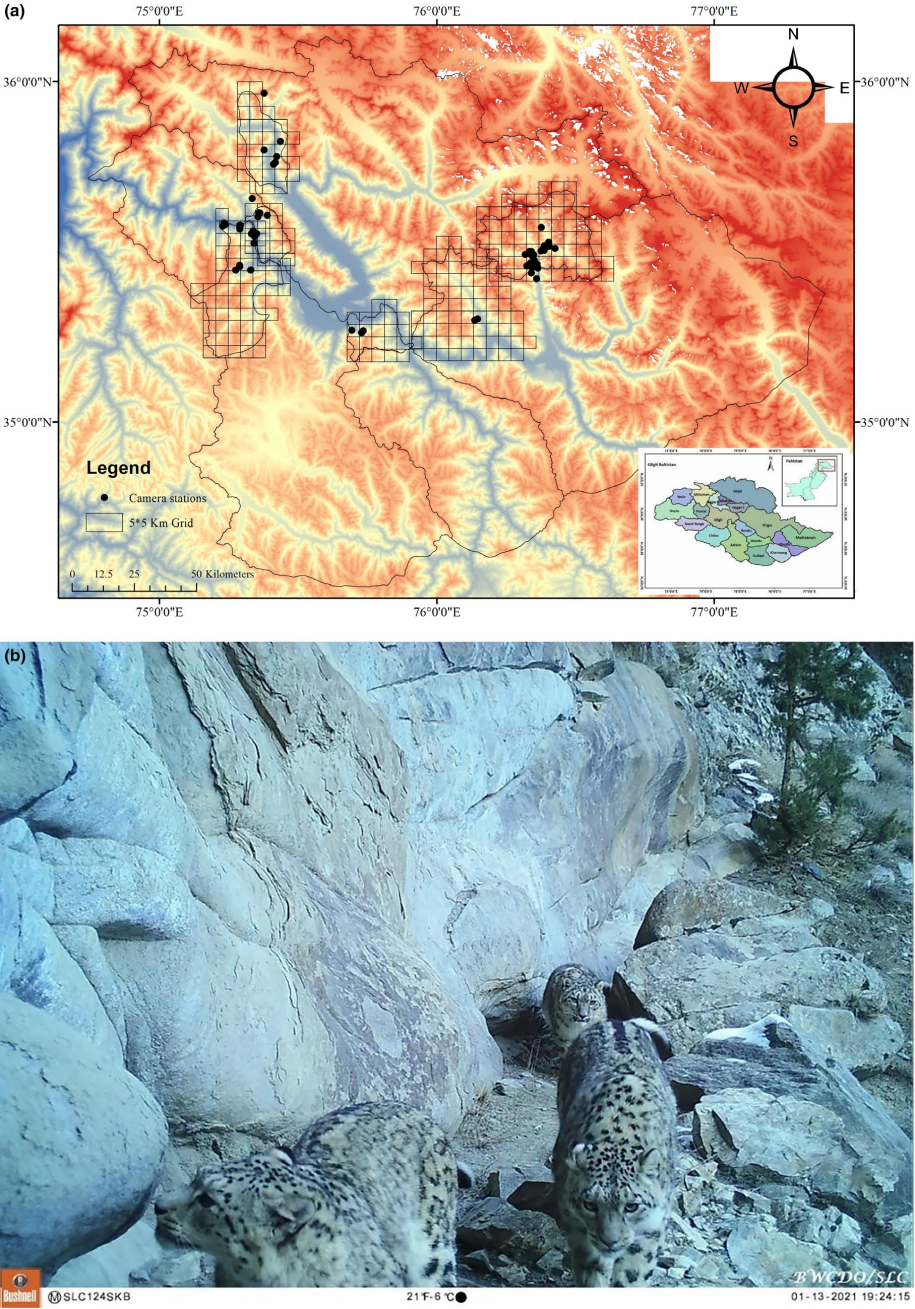
(a) Map showing the camera trapping station in the village conservation community project sites and community controlled hunting areas in Baltistan. (b) The snow leopard is a top predator in the mountain ecosystem of northern Pakistan. Camera traps set up by BWCDO/SLC captured footage in the study sites of SKB Rundo on January 13, 2021.

This study was conducted in fragmented landscapes across four districts: Shigar, Gangche, Skardu, SKB Rondo and Basho in various Community Controlled Hunting Areas (CCHAs) or Village Conservation Community areas (VCCs) (see Figure 1a). Area A, situated in the Saltoro range and Hushe valley, encompasses the villages of Hushe (with 150 households) located in the rugged and broken terrain of the Central Karakoram and Western Himalayan mountain ranges in northern Pakistan (Hussain 2003). Area B, located approximately 70 km west of Skardu city, includes the villages of SKB (200 households) and Basho situated on the south bank of the Indus River housing a small population of Astor markhor (*Capra falconeri falconeri*). Area C covers the mountain ranges to the south and east of Skardu valley incorporating the villages of Shagarthang (Zaman, Jackson, and Hussain 2024; Zaman et al. 2024). Area D lies in the Karakorum mountain range of Basha Shigar valley comprising 166 households with sampling sites located within a mountainous desert with sparse monsoon rains (Zaman et al. 2020).

The average monthly precipitation documented from 2021 to 2022 was 13 mm. In the winter, the area is bitterly cold with the temperature falling below −20°C in the higher altitude parts. The range land is comprised of temperate pastures (Zaman et al. 2020), while the lower parts of the mountain edges were roofed with glacial layers that formed steep slopes and gullies as a result of erosion by snow melt in the spring. Furthermost of the isolated areas were barren lands with rough, broken terrain (Zaman et al. 2019). The vegetation of the area consisted of plant species characteristically found in the high‐altitude desert of Central Asia, such as artemisia (*Artemisia maritima*), ephedra (*Ephedra gerardiana*), wildrose (*Rosa webbiana*), scurbu (*Berberis lyceum*), seabuckthorn (*Hippophae rhamnoides*). The lower slopes were covered with blue pine (*Pinus wallichiana*) forests. The mammalian fauna includes siberian ibex (*Capra sibirica*), marmot (*Marmota caudata*) and Royle's pika (*Ochotona roylei*). Sympatric mammalian predators included red fox (*Vulpes vulpes*), gray wolf (*Canis lupus*), Eurasian lynx (*Lynx lynx*) and (*Panthera uncia*)—see Figure 1b (Zaman, Jackson, and Hussain 2024; Zaman et al. 2024).

### 2.2 Data Collection

#### 2.2.1 Camera Trapping Videos Covering Pre‐Processing

The home range size of snow leopards varies widely ranging from 10 to 35 km^2^ in optimal habitats (Jackson et al. 2006), and 400 km^2^ in marginal habitats with sparsely distributed prey (Zaman, Jackson, and Hussain 2024; Zaman et al. 2024). In high‐quality habitats an estimated minimum home range for an adult female cover approximately two trap stations per 16–30 km^2^. To facilitate data collection, a grid system (5 × 5 km) was adopted (Li et al. 2023), with each grid cell positioned 2 km apart and equipped with one or two infrared cameras (Bushnell and Browning, Shenzhen Weixin Science and Technology Development Co. Ltd., Shenzhen, China) strategically placed to capture images from both sides of visiting snow leopards at trapping stations (Jackson et al. 2006). This camera setup aimed to capture images from both sides of a visiting cat, thereby increasing the likelihood of identifying different individuals from initial capture to recapture (Zhu et al. 2021). However, the camera trap array was less systematic as several grid cells were inaccessible for our team in each year, while a few grid cells had more cameras installed where there were relatively high occurrences of snow leopards; this protocol was followed by Zhu et al. (2021, 2024). Camera site selection prioritized accessibility for monitoring and minimized the risk of damage from natural disasters like avalanches, landslides, floods and soil erosion. Cameras were programmed to record either short videos (30‐s duration) or three photos per trigger event in various CCHAs and VCCs in accordance with the protocol outlined by Zaman et al. (2022).

### 2.3 Camera Trap Station Set‐Up

In order to ensure high‐quality image and video capture, we constructed natural‐looking cairns using locally sourced flat rocks to protect and conceal cameras and their sensors following the methodology described by Jackson et al. (2006). A preliminary test was conducted by walking slowly past the camera trap to activate the sensor establishing the boundaries of the detection zone based on the LED indicator light. By remaining stationary and moving only hands within the cone‐shaped area, we determined the optimal upper and lower detection heights above the ground. Adjustments were made to the sensor height and angle to align the beam at chest height of a snow leopard (35–45 cm) (Jackson et al. 2006). To enhance image quality, all vegetation and obstacles obstructing the cameras were removed as recommended by Zhu et al. (2021). Each video captured was automatically date‐ and time‐stamped along with the corresponding location ID. Between November 2018 and April 2023, a total of 136 camera trap sites were established and active in different CCHAs and VCCs in distinct valleys like Hushe, Skoyo, Basha, Thallay and Hussainabad during each year of study with this extensive effort resulting in a combined trapping duration of roughly 2773 days (Table S1). For more comprehensive details please refer to Table S1. Data collection occurred over five sampling stages in winter seasons. Each camera was visited approximately every 15 days for video or image file download and battery check/replacement (Zhu et al. 2021).

### 2.4 Snow Cover Subtly Impacts Temporal Activity and Marking Behaviors

To begin with, we categorized the winter months into two separate phases, distinguishing between periods with snow‐covered ground and those without based on the duration of recordings from the camera trap stations: one characterized by extreme cold temperatures (−5°C to −27°C) and snow cover from December 25th to February 25th, and the other by warmer temperatures (0°C to 20°C) without snow in November, March and April as described by Zaman, Jackson, and Hussain (2024) and Zaman et al. (2024). Snow cover is known to impact the temporal activity patterns of snow leopards (Johansson et al. 2022; Shen 2020), and other distributed in harsh environments. Secondly, we carefully analyzed 30‐s video clips from different camera traps to pinpoint instances of snow leopards displaying marking and communication behaviors. We divided these behaviors into two time periods distinguishing between when snow leopards were active on snow‐covered ground versus snow‐free ground (Yang et al. 2018). Moreover, the snow leopard marking time and communication behaviour videos were separately recorded during the presence or absence of snow at 30‐minute intervals as described by Zaman et al. (2022). However, we were unable to identify factors such as sex, age classes, or whether the individuals were engaged in mating or non‐mating behaviors. A significant factor influencing the frequency and types of markings made by snow leopards was associated with the onset of the breeding season, as well as the sex and age of the individuals (refer to Ahlborn and Jackson 1986). Moreover, there were a lack of data on seasonal camera trapping in different study areas affected by grazing pressure or pastoral practices (Zaman, Jackson, and Hussain et al. 2024). This differentiation was crucial due to the unique temporal activity patterns of snow leopards compared to other felids, which varied between daytime and nighttime within different seasons (Zaman, Jackson, and Hussain 2024; Zaman et al. 2024).

### 2.5 Spatio‐Temporal Territorial Marking Sites Choice

In every research location, the communication patterns of snow leopards were categorized into nine distinct signals such as scent markings, body rubbing, investigative actions and vocalizations in various environments. The details can be found in Table 1 (Ahlborn and Jackson 1986; Li et al. 2013). The snow leopard marking posts serve as important communication hubs often referred to as “bulletin boards,” where signs are left and read by other leopards. The strategic placement of these bulletin boards is crucial for effective communication (Allen et al. 2016; Li et al. 2013). Our study followed the habitat classification methodology utilized by Ahlborn and Jackson (1986); Li et al. (2013). Each marked site was categorized based on a hierarchical landforms system using descriptors such as terrain type (cliffs, slopes, riverbeds) and feature marked (promontory, cliff face, rock outcrop, refer to Table 1 for details) through camera footage and the Global Position System (GPS) (Ahlbom and Jackson 1986).

**TABLE 1:** Snow leopard behavior and investigation based on camera trap video imagery and recorded feature marked, terrain and habitat types.

Type	Behavior	Definition	References
Scent marking	Scraping	Raking their hind feet through the substrate and then occasionally urinating and/or defecating on the scraped mound of material/or sands (continuous data)	Ahlborn and Jackson (1986)
	Urine spraying	Spraying urine backwards onto overhanging rocks or other vertical surface of rocks (continuous data)	
	Fecal deposition	Defecating; also called “fecal marking” in some studies/scats marks (continuous data)	
Body rubbing	Cheek rubbing	Rubbing their cheek on a rock surface /snags, tree trunks (continuous data)	(Macri and Patterson‐Kane 2011)
	Claw marking	Raking, gouging, or gripping a bushy vegetation or rocks with their claws	
	Rolling	Rolling posterior and onward on the ground; also called “vegetation flattening” in some studies (continuous data)	
	Tail wrapping	Wrapping their tail around a vertical rock surface (continuous data)	
Vocalization	Vocalization	Making breeding calls or other sounds during mating seasons/non‐mating seasons (continuous data)	(Maximilian L. Allen et al. 2016)
Investigating	Olfaction	Sniffing to examine cues and signals, distinguished by the snow leopard's nose within one head length of a scrape or other cue; also called “sniffing” in some studies (continuous data)	Li et al. (2013)
	Flehmen response	Lifting head and curling back upper lip, sometimes arching neck backwards, in order to expose vomeronasal organ (continuous data)	Allen, Wittmer, and Wilmers (2014)
Feature marked	Cliff face or base (0), rock outcrop or boulder (1), (binary variable)		Ahlborn and Jackson (1986)
Terrain type	Distinctly broken slopes (0); moderately broken slopes (1), (binary variable)		Ahlborn and Jackson (1986)
Habitat types	Bare area (75% open area) (0); herb/shrubs mixed land (75% dominated by herbaceous or shrubs) (1), (binary variable)		Zaman, Jackson, and Hussain 2024; Zaman et al. 2024

### 2.6 Relative Abundance Indices (RAI)

Secondly, for two distinct periods (the presence or absence of snow) of winters in each study site, these temporal marking activities were grouped into three categories based on time‐ or date‐stamped on each video with camera Identity Number: (1) nocturnal, with activity mainly between 1 h after sunset and 1 h before sunrise; (2) diurnal, with activity primarily between 1 h after sunrise and 1 h before sunset; and (3) the crepuscular periods, occurring 1 h before sunrise to 1 h after sunrise and 1 h before sunset to 1 h after sunset (Zhao et al. 2020). Following this, we recorded the content viewed at each camera location individually for each instance of snow cover as described earlier in every year under examination (Zaman et al. 2022). The number of videos along with the total number of camera functioning days were recorded at each site during the monitoring period. To quantify the RAI of snow leopards temporal marking activity events during the day or night at each trap site in the presence or absence of snow in different habitats, we calculated the index in units of camera trap days following the methodology described by Zhu et al. (2024). The RAI for a specific marking event was calculated using the formula: RAI = (Ni/TRAPDAYi) × 100, where Ni represents the number of independent valid videos of marking events I, and TRAPDAYi indicates a camera working day. Each camera trap station was treated as a distinct spatial point to analyze temporal activity patterns and the frequency of various communication behavior detection occurrences during the day or either night of snow leopards based on video records (Li et al. 2013; Zhao et al. 2020).

### 2.7 Data Analysis

In this study, we investigated how the peak timing of snow leopard marking events varies between day and night based on the presence or absence of snow cover. This comparison was done by fitting non‐parametric kernel density functions using the “Overlap” package with default bandwidth parameters as recommended by Johansson et al. (2022); Li et al. (2023). Circular density curves were used to compare the patterns and the coefficient of overlap (∆1 and ∆5) was calculated ranging from 0 (no overlap) to 1 (complete overlap) following the method proposed by Johansson et al. (2022); Yang et al. (2018). We estimated the overlap coefficient (∆1 and ∆5) for the temporal marking activity of snow leopards in the presence or absence of snow cover and during day versus night events for each communication behavior. Video analysis was performed at a minimum time interval of 30 min to avoid pseudo replication as suggested by Kays et al. (2020); Zhu et al. (2021). Each camera trap was independently assessed as a spatial point to determine snow leopard marking time and communications behavior records occurring between sunset and sunrise—precise times vary slightly throughout the year based on the distance from the equator and seasons. To adjust for these variations in daylight hours throughout the year (Johansson et al. 2022), we utilized the “sunTime” function of the “overlap” package version 0.3.2 in R (statistical software V.3.5.1, www.r‐project.org) to convert times to radians for analysis (please refer to Johansson et al. (2022) for detailed information). We assessed the range of individuals using the same site and the visitation rate between consecutive visits to investigate snow leopard variability in their temporal activities across different communication behaviors and habitat types. Subsequently, we examined significant variations in the marking time events of snow leopards in areas with and without snow cover for day versus night by using Watson's 2‐sample test in the “circular (version 0.4‐94)” package in R 4.0.5.

### 2.8 Model Selection

In the second phase of our research, we investigated how snow cover affects the daily activity of snow leopards and also examined whether snow leopards exhibited a preference or avoidance for marking activity during day or night using logistic generalized linear models (GLMs) with multiple explanatory variables (Zaman et al. 2020). Initially, we used the Pearson correlation test to evaluate multicollinearity in Global Models 1 and 2 in order to validate the precision of our models (Zaman et al. 2020). Any covariates with absolute correlation coefficients exceeding 0.7 with other covariates were removed to address potential correlation issues (Zaman, Jackson, and Hussain 2024; Zaman et al. 2024). For instance, covariates like olfaction (*r* = 0.91), terrain types (*r* = 0.82) and habitat type (*r* = 0.87) were identified as highly correlated in the absence of snow, while features marked (*r* = 0.11) and terrain types (*r* = 0.80) were found to have highly significant correlations in the presence of snow. For example, Alhborn and Jackson (1986) observed that snow leopard were attracted to certain local features when marking (e.g, overhanging boulders for scent spraying; sandy soil for scraping etc. Consequently, these variables were eliminated from the final model to ensure the accuracy of our results (Zaman, Jackson, and Hussain 2024; Zaman et al. 2024). To determine the accuracy of classification models for activities during night and day events, we utilized ROC analysis with a user‐defined threshold value of 0.5 using packages like rocr, lmtest and car (Zaman, Jackson, and Hussain 2024; Zaman et al. 2024) and selected models with an AUC score of ≥ 0.7 (Zaman, Jackson, and Hussain 2024; Zaman et al. 2024). Furthermore, we assessed the data normality by conducting a Shapiro–Wilk test using the ‘mvnormalTest’ package. The best model was chosen based on criteria such as AICc, delta AIC_C_ and weights using lme4 and MuMIn packages in R (Zaman, Jackson, and Hussain 2024; Zaman et al. 2024). In assessing the significance of parameters, we considered their levels alongside standard errors (SE) and 95% confidence intervals (CI) (Hua et al. 2020).

For Model 1, we employed GLMs to classify the timing of marking activity as either day or night in the absence of snow (Nakazawa 2022). Six covariates were considered and four GLMs candidate models were run to assess how these factors influenced the time spent on marking activities during day versus night as used by Ridout and Linkie (2009). In the analysis of the second model (Model 2), we examined how the presence of snow impacted marking time using a binary response variable. The model included seven covariates and three GLMs candidate models were developed to analyze the presence of snow and its effects on the marking activity patterns during day versus night (Zaman, Jackson, and Hussain 2024; Zaman et al. 2024), and all analyses were computed in R (Maximilian L. Allen et al. 2016; Harmsen et al. 2010).

### 2.9 Differences in Marking Time and Communication Patterns Between Day and Night

We assessed the interaction between the presence and absence of snow and their impact on the detection frequency of different communication behaviors of snow leopards during both day and nighttime (Prugh and Golden 2014). For our analysis, we conducted pairwise comparisons between day and night marking time along with various communication behaviors in relation to the presence or absence of snow cover using the Mann–Whitney *U* test (Li et al. 2023). These comparisons assessed the variation in the independent detection frequencies of each communication behavior, while considering day and night marking time events as grouping variables and the analysis included Mann–Whitney *U* tests with a statistically significant threshold level set at *p* < 0.05 (Allen et al. 2016).

## 3 Results

### 3.1 Relative Abundance Indices and Occurrences of Snow Leopard Temporal Marking Events

**FIGURE 2:**
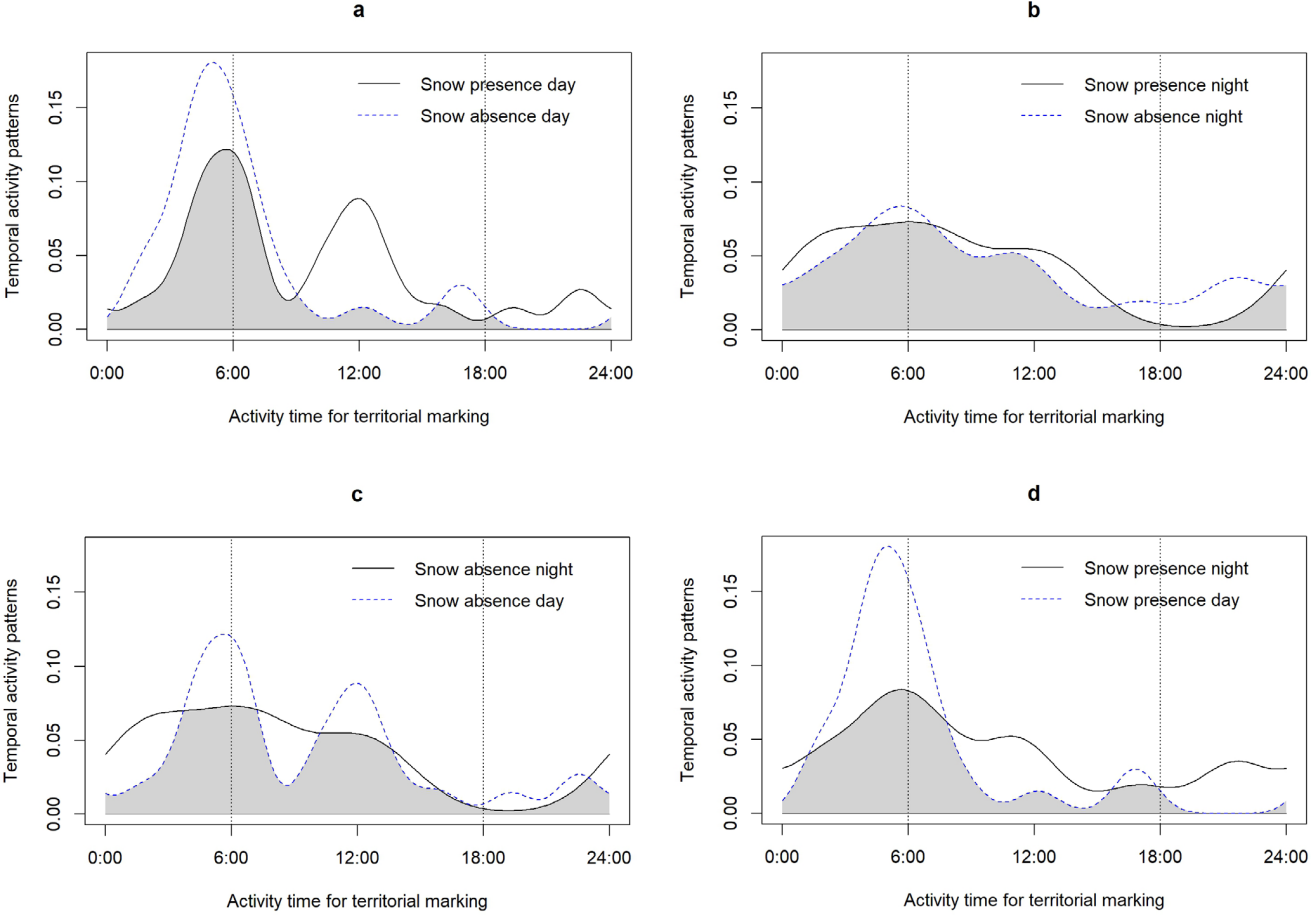
Snow leopard temporal territorial marking time events in northern Pakistan Baltistan regions, comparing (a) snow presence versus snow absence day marking activities, (b) snow presence night versus snow absence night marking activities, (c) snow absence day versus night marking activities and (d) snow presence day versus night activities—day and crepuscular periods were combined in the analysis.

During the study period, the cameras captured 813 distinct sightings of snow leopards over a sampling period of 2773 trap days (see Table S1). The data set contained 103 records of snow leopards marking their territory through videos and 40 of these events took place in snowy conditions, while the remaining 63 sightings occurred in snow‐free environments (Tables 1 and 2). The RAI value for marking activity in open areas without snow during the nighttime was notably higher at 15.53 compared to 3.84 when snow was present; a similar trend was also noticed in herb/shrub dominated areas. In particular, the RAI was found to be highest for twilight activities in snowy conditions and for nocturnal activities in the absence of snow during the winter season (Table 2). Marking events were predominantly observed in open areas and were least frequent in mixed herb/shrub‐type habitats (refer to Table 2). Moreover, utilizing the kernel density function revealed how the presence or absence of snow cover affects the activity patterns favoring or avoiding by snow leopards. Marking behaviors were noted both in the presence and absence of snow during daytime, with higher activity levels during daylight hours when snow was present, especially around 06:00 and 12:00 hours. The Watson test revealed non‐significant differences in the daily marking activity patterns of snow leopards between snowfall and snow‐free conditions during both daytime (Δ = 0.62, *p* = 0.60) and nighttime (Δ = 0.41, *p* = 0.30) periods. In contrast, leopards exhibited twilight activity peaks around 06:00 and 18:00 hours in snow‐free conditions (Figure 2a). Nighttime marking activities peaked between 06:00 and 18:00 in snowy areas, whereas in the absence of snow, they peaked around 06:00 and 24:00 (Figure 2b). In snow‐free conditions, marking time activities overlapped during both day and night from 06:00 to 12:00, with daytime marking peak at midday (Figure 2c). On the other hand, the presence of snow resulted in marking activities peaking in the evening and midnight (Figure 2d). Additionally, the absence of snow did not significantly affect the marking behavior of snow leopards between day and night (Δ = 0.74, *p* = 0.22) leading to a notable contrast in marking activities between snowy daytime and nighttime (Δ = 0.63, *p* < 0.01; Table 3).

**TABLE 2:** The number of video events and relative abundance index (RAI per 100 camera trap days) for snow leopards during the presence and absence of snow in various study sites in Northern Pakistan.

Winter seasons	Presence of snow			Absence of snow		
Habitat types	Crepuscular	Diurnal	Nocturnal	Crepuscular	Diurnal	Nocturnal
Bare area	12 (11.65)	3 (2.91)	8 (7.76)	7 (6.79)	13 (12.62)	16 (15.53)
Herbs/Shrubs	8 (7.76)	3 (2.91)	6 (5.82)	3 (2.91)	6 (5.82)	18 (17.47)
Total for each events	20 (19.41)	6 (5.82)	14 (13.59)	10 (9.70)	19 (10.67)	34 (33.09)
Total captured	103					

**TABLE 3:** The comparison significances between day versus night marking time events of snow leopards in study areas with the presence of snow and without snow, utilizing the Watson's 2‐sample test and overlap for day‐night activity patterns (∆1 or ∆5) and confidence interval (CI).

Comparison snow covers	Activities events	(∆1 or ∆5; cl)	Watson's test (*W*, *p* < 0 05)
Snow presence vs. absence day	Snow presence (29) and absence (26)	0.62 (0.44–0.79)	*W* = 0.44, *p* = 0.60
Snow presence vs. absence night	Snow presence (34) and absence (14)	0.83 (0.66–1.00)	*W* = 0.24, *p* = 0.32
Snow absence night vs. absence day	Snow absence night (34) and day (29)	0.74 (0.59–0.88)	*W* = 0.10, *p* = 0.22
Snow presence night vs. presence day	Snow presence night (14) and day (26)	0.63 (0.41–0.86)	*W* = 0.40, *p* < 0.01

### 3.2 The Selection of Marking Sites Between Day and Night‐Time

The seasonal occurrence (month of year) of snow leopard during in the snow absence days, three nighttime scent marking behaviors were documented: scraping (9 visits), urine spraying (7 visits) and scat deposition (5 visits). Snow leopards were observed to typically make one scrape during scraping events (90% of the time), with a rare instance of an individual creating two adjacent scrapes. In habitats with bare land, olfaction and scraping were more prevalent, while fecal deposition increased in areas with mixed herb and shrub vegetation (see Figure S1a). These scent markings were commonly found near cliff rock faces or bases, rock outcrops and boulders.

During the daytime, scraping (3 visits), urine spraying (10 visits) and scat deposition (5 visits) were recorded (refer to Figure S1b). Urine spraying and olfaction were predominantly associated with rock outcrops on bare/barren land, while scraping was more commonly linked to slopes that were distinctly or moderately broken in areas with bare land or mixed herb and shrub vegetation. Two distinctive body rubbing behaviors were observed in the absence of snow: cheek rubbing (2 visits) and tail wrapping (3 visits), which were observed to occur more frequently during the daytime in habitats with bare land.

Investigative behaviors included olfaction (10 visits) and flehmen response (1 visit) with olfaction being more commonly observed than flehmen response during nighttime. Daytime olfaction was recorded at 5 visits with no instances of vocalization being documented. When snow cover was present at nighttime, two scent marking behaviors were observed: scraping (7 visits) and urine spraying (2 visits) (see Figure S1c). Snow leopards were found to usually create one scrape during scraping events (95% of the time), with a few cases of an individual making two adjoining scrapes. In the daytime, scraping (4 visits), urine spraying (2 visits) and scat deposition (2 visits) were documented. Two body rubbing behaviors were also noted during the presence of snow: cheek rubbing (2 visits) and tail wrapping (1 visit), primarily associated with slopes that were distinctly or moderately broken with bare land or mixed herb and shrub vegetation and prominently showcasing features such as rock outcrops, cliff faces and tuft of grasses. Cheek rubbing was more prevalent than tail wrapping with both behaviors being more common during the daytime (refer to Figure S1d). There was one investigative behavior recorded: olfaction (2 visits), with daytime olfaction found in 7 visits and vocalization documented in 3 visits during the daytime.

In the absence of snow, three independent variables namely scraping, urine spraying and body rubbing were the significant marking activities (*n* = 4, *ώ* = 1; Table 4). Fecal deposition was the second most explanatory model/variable (*K* = 5, *ώ* = 0.40). Daytime activities favored scraping and urine spraying compared to nighttime but body rubbing was not significant (Table 5). In the presence of snow, the marking activities were best explained by four independent variables: scraping, urine spraying, body rubbing and olfaction (*n* = 5, *ώ* = 1; Table 6, Figure S2a–l). The second model selected fecal deposition as significant (*K* = 6, *ώ* = 0.36), while the third model integrated habitat (*K* = 7, *ώ* = 0.08; Table 6). Scraping during the day and night revealed a tendency to avoid certain patterns (Table 7; Figure S2i,j), whereas it was noted that snow leopards exhibited a preference for specific behaviors during the timing of their marking activities. These behaviors included urine spraying, body rubbing, and scent marking, which emerged as significant methods for communication among individuals.

**TABLE 4:** Summary of candidate models for generalized linear model with delta ∆AICc < 2, investigating the difference between diurnal and nocturnal marking time events of snow leopard with explanatory variables in the absence of snow, AIC_C_ represents Akaike's information criterion adjusted for small sample sizes; *K* denotes degrees of freedom; *W*_*i*_ signifies Akaike weight.

Response ~ explanatory variables absence of snow cover	log Lik	*K*	AICc	∆AICc	*W*_*i*_
(a) Marking time ~ scraping + urine spray + body rubbing	−19.02	4	48.05	0.00	1.00
(b) Marking time ~ scraping + urine spray + fecal deposition + body rubbing	−18.92	5	49.85	1.79	0.40
(c) Marking time ~ scraping + urine spray + body rubbing + feature mark + fecal deposition	−18.87	6	51.74	3.68	0.15
(d) Marking time ~ scraping + urine spray + fecal + body rubbing + features mark	−18.49	6	54.98	6.92	0.03

**TABLE 5:** Parameter estimates from the most/best fitting snow leopard models, along with their standard errors (SE) and 95% confidence intervals (CI), exponent coefficient are presented. The asterisk (*) denotes model parameters with a significant impact on diurnal versus nocturnal marking time events in the absence of snow, which is associated with various explanatory variables.

Parameter	Estimate	S.E.	exp(coef)	*z* Value	Lower	Upper
Intercept	−0.79	1.40	−0.16	−0.27	0.00	1.91
Scraping	−0.18	0.87	−0.30	−0.34	0.04	1.61*
Urine spray	−2.03	1.35	−7.65	−1.50	0.73	2.48*
Body rubbing	−1.44	1.38	−4.25	−1.04	−0.34	1.71

**TABLE 6:** Summary of potential generalized linear model candidates (∆AICc < 2) exploring the marking time events of snow leopard during daytime versus nighttime in snowy conditions, utilizing data across various explanatory variable. AIC_C_ is Akaike's information criterion adjusted for small samples; *K* represents degrees of freedom; *W*_*i*_ stands for Akaike weight.

Response ~ explanatory variables presence of snow cover	log Lik	K	AICc	∆ AICc	*W*_*i*_
(a) Marking time ~ scraping + urine + rubbing + olfaction	−16.85	5	47.64	0.00	1.00
(b) Marking time ~ scraping + urine + fecal + rubbing + olfaction	−17.32	6	48.65	1.00	0.36
(c) Marking time ~ scraping + urine + fecal + rubbing + olfaction + habitat	−17.82	7	51.70	4.05	0.08

**TABLE 7:** Parameter estimates from the most/best fitting snow leopard models, along with their standard errors (SE) and 95% confidence intervals (CI), exponent coefficient are presented. The asterisk (*) denotes model parameters with a significant effect on diurnal versus nocturnal temporal marking activity in the presence of snow, which is related to the explanatory variables.

Parameter	Estimate	S.E.	exp(coef)	*z* Value	Lower	Upper
Intercept	−0.10	0.96	0.90	−0.10	−2.06	1.90
Scraping	−2.21	1.20	0.10	−1.83	−4.93	−0.07*
Urine spray	−0.36	1.34	1.43	−0.26	0.73	2.48*
Body rubbing	−2.35	1.18	10.49	−1.99	0.34	1.71*
Olfaction	−2.34	1.19	10.481	−1.96	0.36	3.19*

**FIGURE 3:**
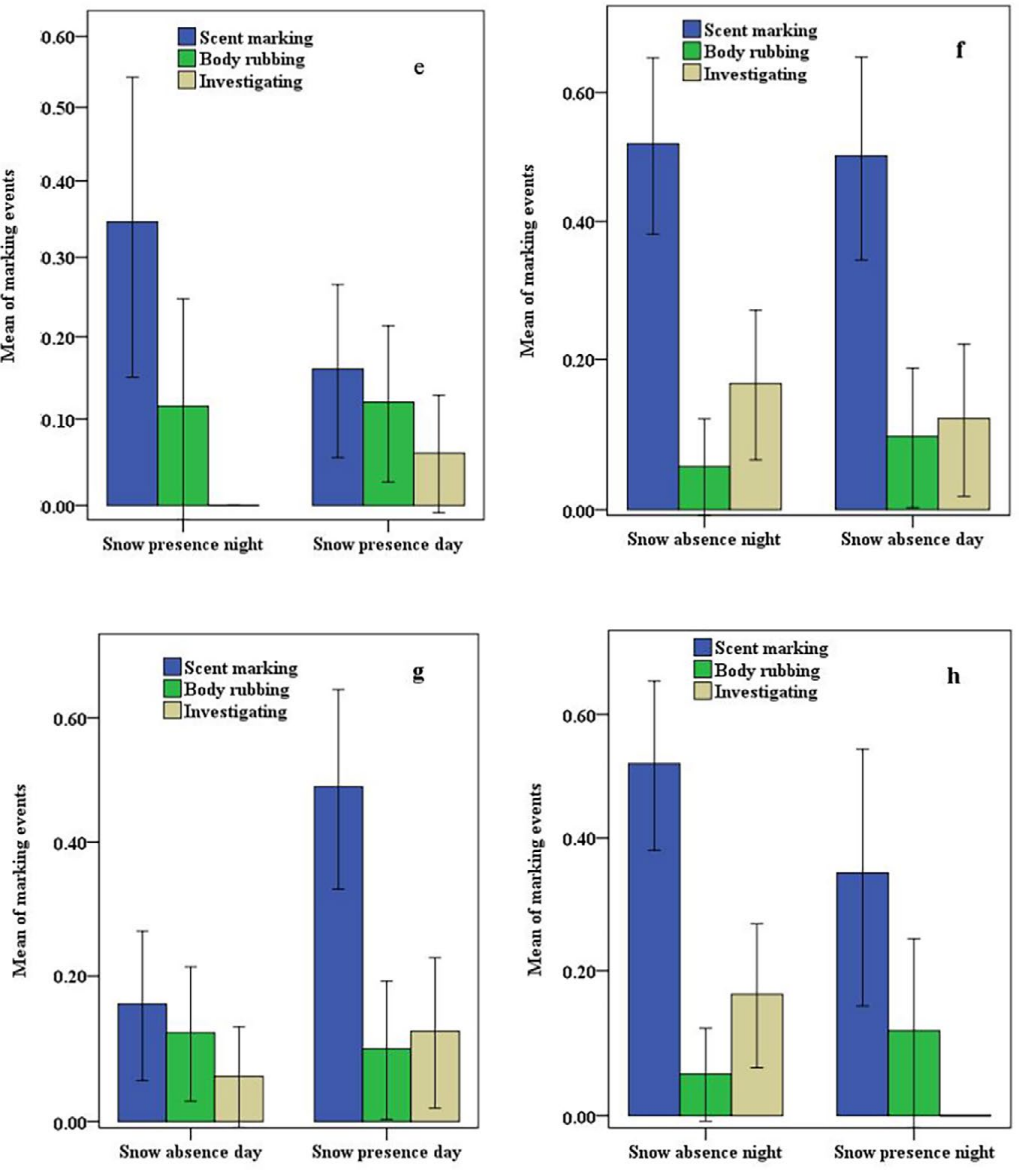
The standard error means difference in the three distinct communication behaviors (scent marking, body rubbing and investigating) in snow leopards during day versus night for (e) presence of snow day and night, (f) absence of snow day and night, (g) snow absence versus presence day and (h) snow absence versus snow presence night.

A comparison of communication behaviors between day and night time marking in the absence of snow showed no significant differences in scent marking (*U* = 111.00; *p* = 0.85), body rubbing (*U* = 108.00; *p* = 0.46) and investigating (*U* = 103.01; *p* = 0.51). Similarly, in the presence of snow, there were no significant differences in scent marking (*U* = 529.00; *p* = 0.06), body rubbing (*U* = 998.00; *p* = 0.95) and investigating (*U* = 962.00; *p* = 0.20). It is worth noting that scent marking significantly differed for snow leopards in the absence versus presence of snow during the day (*U* = 689.00; *p* < 0.00), while investigating behavior showed a significant difference at night (*U* = 585.00; *p* < 0.02) (refer to Table S2 and Figure 3 for more details).

## 4 Discussion

### 4.1 A Novel Approach Using Camera Traps Techniques

In the present study, we analyzed long‐term camera trapping data of snow leopards using the method established in previous works by Jackson et al. (2006); Li et al. (2023). Our research delved into new findings that address a significant gap in understanding the temporal activity and communication behaviors and observations of free‐roaming snow leopards in northern Pakistan. This study highlights the importance of understanding the spatial and temporal distribution patterns of snow leopards in different territorial locations and these spatio‐temporal occurrence areas can serve as vital tools for conservation efforts helping to prioritize actions aimed at protecting their natural habitats (Buzzard, Li, and Bleisch 2017; Fox et al. 2024). To date, there is a lack of detailed research in existing literature on the territorial marking behaviors of snow leopards originating from Pakistan. In our research, we concentrated solely on the presence or absence of snow at camera trap locations during the winter months when snow leopards were observed. Our study did not address variations in territorial marking between mating and non‐mating periods, nor did it investigate behaviors across different seasons, ages, or sexes, primarily due to insufficient data resulting from limited seasonal camera trapping (Zaman, Jackson and Hussain et al. 2024). While our analysis was restricted to winter, it is important to recognize that the seasonal age structure and sex ratio of the study population play a crucial role in shaping social interactions (Jackson and Ahlborn 1989). Former studies have indicated that the marking behaviors of snow leopards in captivity are influenced by artificial constraints and management protocols, thereby limiting their comparability to wild populations (Ahlborn and Jackson 1986; Mellen 1993). Research conducted by Ahlborn and Jackson (1986); Mallon (1984) provided detailed descriptions of snow leopard marking behavior in Nepal, Ladakh and Mongolia (McCarthy and Munkhtsog 1997). However, a significant knowledge gap remains when it comes to understanding how snow leopards spatio‐temporal behave in the presence or absence of snow cover for both day and night (Johansson et al. 2021). Our observations indicated that various behavioral traits and communication patterns were significantly affected by the time of day versus night—and the presence of snow. Specifically, behaviors such as tail wrapping, vocalizations, the flehmen response, cheek rubbing and scat deposition showed a clear bias related to these environmental variables. However, the scope of our findings was constrained by insufficient data and challenges in observation during certain times. To gain a deeper understanding of these patterns and their implications for the species behavioral ecology, further research with more comprehensive data collection is essential (Ahlborn and Jackson 1986; Mellen 1993).

### 4.2 Relative Abundance Indexes

We found that the RAI for the marking activity of snow leopards in bare areas without snow at night was higher compared to the presence of snow. This observation was also noted in herb/shrub areas, which deviates from a previous study characterizing snow leopards as shy solitary hunters with crepuscular activity patterns that change seasonally from dusk to dawn with increasing temperatures (Johansson et al. 2022). Additionally, the RAI for both crepuscular and nighttime activities demonstrated peak levels regardless of the snow conditions throughout the winter season, a trend also evident in species such as the North China leopard (*Panthera pardus japonensis*) (Zaman et al. 2022), Amur tiger (*Panthera tigris altaica*) and Amur leopard (*Panthera pardus orientalis*) (Yang et al. 2018).

We found that marking activities predominantly took place in dry habitats situated above the tree line. These areas characterized by their rugged terrain and elevated ridges, as well as the bases of cliffs, served as the primary locations for such behaviors and our results are similar to previous research conducted on common leopards (*Panthera pardus*) (Buzzard, Li, and Bleisch 2017). During our field visits, we observed that many ungulates also favored bare areas but only during the daytime (Zaman, Jackson, and Hussain 2024; Zaman et al. 2024). It is predicted that snow leopards exhibit their activity during the peak active times of their prey (Li et al. 2023). In the northern regions of Pakistan, mountain ungulates serve as the primary food source for snow leopards (Anwar et al. 2011; Hacker et al. 2021).

Our findings reveal significant differences in the scent marking behaviors of snow leopards on snowy versus snow‐free winter days. However, it is important to note that our investigation was confined to winter periods captured by camera traps (Zaman, Jackson, and Hussain 2024). Previous research suggests that many felid species exhibit seasonal variations in their marking rates (Ahlborn and Jackson 1986), particularly between winter (during snowfall) and autumn or summer (Maximilian L. Allen et al. 2016). This aligns with prior research indicating that snow leopards adapt their marking behavior according to the time of day potentially in response to changing climatic conditions or habitat threats (Li et al. 2016; Liu et al. 2022). We also detected that crepuscular activity peaked around dawn and dusk, with these results supporting the prediction that predators should reduce or increase activity at times when major prey species are active (Liu et al. 2022; Prugh and Golden 2014). We observed that nighttime marking activities peaked around twilight. Other studies also revealed that felids avoided human disturbances and shifted their activity at night to reduce conflicts (Yang et al. 2018).

Explicitly during the presence of snow, the marking activities of snow leopards showed a peak in the evening and at midnight. It is anticipated that these prominent peaks in activity are linked to the movement and hunting patterns of snow leopards during both day and night, presenting increased concealment opportunities in habitats with mixed herbs or shrubs and undisturbed trails which also serve as a platform for territorial marking (Macdonald and Loveridge 2010). Literature sources support several of our findings regarding the behavior of snow leopards (Mori et al. 2021). For instance, it is suggested that snow leopards adjust their daily activity patterns being more active during sunset and sunrise in summer and sunrise and sunset in winter, in order to regulate their body temperature in harsh environments (Johansson et al. 2022; Stokes, Slade, and Blair 2001).

Our findings indicate that the lack of snow did not significantly influence the marking behavior of snow leopards during both daytime and nighttime observations. Nevertheless, a notable disparity in marking activities was observed between day and night when snow was present. Others have also revealed that animals are considered thermoneutral when they do not actively regulate their body heat (Shen 2020). While the thermoneutral range for snow leopards is currently unknown, other medium‐sized and large felids less adapted to cold climates have a thermoneutral range of 9°C to 37°C (McNab 2000). In contrast, the Arctic fox (*Vulpes lagopus*), well‐adapted to polar conditions remains thermoneutral down to −7°C with its winter coat and at 5°C in summer (Fuglesteg et al. 2006).

### 4.3 Marking Behavior Changes With Snow Cover and Habitat Features Vary Between Day and Night

Our observation revealed that the communication patterns of snow leopards were influenced by the time of day. During the absence of snow, we noted scraping behavior with a clumped or relict scrapes being the typical behavior (Ahlborn and Jackson 1986). Interestingly, there was an instance where an individual made two adjacent scrapes—a behavior observed in sunda clouded leopards (*Neofelis diardi*) in Indonesia (Allen et al. 2016). In habitats with bare land, we found that olfaction and scraping were more common, whereas in areas with a mix of herbs and shrubs, fecal deposition increased. Previous studies have suggested that these sites may act as markers for many species living in the same area (Harmsen et al. 2010; Vogt et al. 2014), potentially helping to reduce conflicts by segregating activities at different times (Li et al. 2013).

Throughout the day, we noticed that snow leopards exhibited a higher inclination towards urine spraying and olfaction in the vicinity of cliffs, rock formations and large rocks found in open land habitats. Scraping actions were correlated with rugged slopes, ranging from distinct to moderate aligning with existing research (Ahlborn and Jackson 1986; Allen, Wittmer, and Wilmers 2014). Our findings showed that snow leopards preferred areas with bare habitats where communication behaviors were more prevalent, potentially due to improved prey detection and reduced grazing pressure compared to areas with a mixed vegetation cover, as seen in felids in Nepal (Ahlborn and Jackson 1986), and Tibet China (Li et al. 2013). Sniffing behaviors were linked to broken slopes suggesting that snow leopards favor high mountain ranges with rocky, steep terrain and ridges for marking activity (Sunquist and Sunquist 2017). We also detected that snow leopards exhibit marking behavior both in the presence and absence of snow with a preference for marking spots located under rocky outcrops or cliffs regardless of the time of day or night. Studies have indicated that these majestic cats adjust their activity patterns according to the season (Johansson et al. 2022; Zaman et al. 2022). Research on other big cats like jaguars (*Panthera onca*) and pumas (*Puma concolor*) (Harmsen et al. 2010), as well as tigers (*Panthera tigris*) (David Smith, McDougal, and Miquelle 1989) has revealed that areas where communication behaviors are displayed are frequently visited by multiple individuals.

During the day, we noticed that urine sprays were commonly found near cliffs, rock outcrops and boulders in open areas, while cheek rubbing behavior was more frequent on slopes that were moderately broken. These findings shed light on the behavioral patterns of snow leopards in different environmental conditions (Li et al. 2013; Vogt et al. 2014) and other solitary felids (du P. Bothma and le Richet 1995; Ghoddousi et al. 2008). The utilization of diverse communication behaviors in snow leopards is consistent with observations in other Panthera species as highlighted by Allen et al. (2016).

Our study found that the marking behavior of snow leopards, whether nocturnal or diurnal varied in response to habitat conditions when snow was present or absent. Specifically, during daytime with snow cover, snow leopards showed a preference for using urine spray marks and relying on olfaction for communication, a pattern consistent with observations in other felids (Allen et al. 2016; David Smith, McDougal, and Miquelle 1989). In contrast, scraping occurred less frequently or at lower rates during day (and night) when the ground was covered by snow that also hides existing marks. Ahlborn and Jackson (1986), demonstrated that some 70%+ of sites with visible scrapes were repeated remarked, I,e freshened up. We predicted that these significant variables in the marking behavior of snow leopards are linked to the movement patterns and hunting preferences as shown in Persian Leopard (*Panthera pardus saxicolor*) (Ghoddousi et al. 2008); conversely, in the absence of snow during the daytime, snow leopards exhibited a preference for using scrape marks and urine spray. We assumed that this shift in behavior could be related to the avoidance of snowy conditions or may increase activity as they move towards a safe or warmer refuge (Li et al. 2023).

Furthermore, our investigation discovered that cheek rubbing and urine spraying were commonly observed in all habitat types, but vocalization behaviors were infrequently observed at our research sites, primarily due to the limited sample size that remained unexamined. In prior research, it was observed that vocalizations during mating particularly from elevated and rugged locations play a significant role in facilitating mate attraction and identification among cats. This finding underscores the importance of vocal communication in the mating behaviors of felines. (Maximilian L. Allen et al. 2016). It has been proposed that carnivores utilize sign posts to reduce energetic costs (Alberts 1992), and these marking posts can be likened to bulletin boards where signals are posted and read (White, Swaisgood, and Zhang 2003). Snow leopards leave various types of markings along their travel routes, which may serve as a form of social communication through visual or olfactory cues. These signs include scrapes, scent spraying, scat deposition, claw raking, urination and tracks (McCarthy and Chapron 2003). Scrapes are “V” shaped marks created by snow leopards raking the substrate (soil, snow, shale, vegetation) with their hind legs sometimes accompanied by urine and scat (Ahlborn and Jackson 1986).

## 5 Conclusions and Conservation Implications

The majestic snow leopards, a rare and endangered species of large cats indigenous to Central and South Asia (Hacker et al. 2021), are facing a growing threat due to conflicts between humans and wildlife particularly in Baltistan, Pakistan (Zaman, Jackson, and Hussain 2024; Zaman et al. 2024). To combat this challenge, conservationists have been employing innovative strategies such as predator‐proofed enclosures and insurance programs to safeguard snow leopards (Bulte and Rondeau 2007; Hussain 2000). While some studies have explored the functional significance of marking patterns in snow leopards and the qualitative characteristics of their preferred habitat for marking, there has been a lack of quantitative analysis on the specific attributes of their chosen marking sites at a more detailed level (Ahlborn and Jackson 1986). In this study, we delved into the selection of marking sites of snow leopards, particularly related to scraping and scent‐spraying behaviors during the presence and absence of snow by using camera traps (Jackson et al. 2006). Our findings revealed new insights into the marking patterns during both day and night, considering the influence of climatic conditions. These marking sites could serve as valuable tools for conservation efforts (Hacker et al. 2021). Our research also suggested that there is a need for further exploration into gender‐specific identification methods for marking sites and conducting population assessments through genetic studies of snow leopards, which would aid in the development of comprehensive long‐term conservation strategies (Vogt et al. 2014).

## Author Contributions

**Muhammad Zaman:** investigation (equal), methodology (equal), writing – original draft (equal). **Yi Chen:** supervision (equal). **Rodney Jackson:** funding acquisition (equal) and editing of final paper, writing – review and editing (equal). **Shafqat Hussain:** project administration (equal), supervision (equal).

## Conflicts of Interest

The authors declare no conflicts of interest.

## Supporting information


Data S1.

## Data Availability

The data input for the model and the R script provided as additional Supporting Information for the manuscript.
