# Supplementary material for: Silent Signals in the Snow: Tracking the Spatio‐Temporal Territorial Marking Behavior of Snow Leopards (Panthera uncia) in the Mountainous Region of Baltistan, Pakistan
Source: Ecol Evol. 2024 Dec 11;14(12):e70518. doi: 10.1002/ece3.70518 (PMC11634815; doi:10.1002/ece3.70518)
Supplement: Supplementary file 1 — Data S1. [file ECE3-14-e70518-s001.zip › SUppl_SL-final_figures_and_tables_24.docx]

| Name of Village | Years of Study | Duration (days) | Camera Stations (number) | Leopards Detection (number) |
| --- | --- | --- | --- | --- |
| Basha | 2018 to 2019 | 123 | 8 | 42 |
| Skoyo | 2018 to 2019 | 91 | 11 | 142 |
| Hussainabad | 2018 to 2019 | 50 | 3 | 0 |
| Basho | 2019 to 2020 | 179 | 2 | 16 |
| Krabathang | 2019 to 2020 | 92 | 10 | 45 |
| Hushe | 2019 to 2020 | 157 | 20 | 95 |
| Skoyo | 2020 to 2021 | 100 | 4 | 111 |
| Krabathang | 2020 to 2021 | 96 | 8 | 169 |
| Mendi | 2020 to 2021 | 71 | 3 | 0 |
| Hushe | 2020 to 2021 | 192 | 8 | 40 |
| Khumerah | 2020 to 2021 | 123 | 3 | 1 |
| Skoyo | 2021 to 2022 | 201 | 8 | 36 |
| Krabathang | 2021 to 2022 | 192 | 16 | 26 |
| Sultanabad Basho | 2021 to 2022 | 142 | 2 | 0 |
| Khumerah | 2021 to 2022 | 213 | 3 | 0 |
| Thallay | 2021 to 2022 | 100 | 3 | 5 |
| Hushe | 2021 to 2022 | 151 | 6 | 18 |
| Skoyo | 2022 to 2023 | 145 | 5 | 44 |
| Mendi | 2022 to 2023 | 123 | 3 | 5 |
| Hushe | 2022 to 2023 | 100 | 6 | 15 |
| Thallay | 2022 to 2023 | 132 | 4 | 3 |
| Total | 5 years (21 times) | 2773 | 136 | 813 |

Table S1: Monitoring Snow Leopard Detection Occurrence through Camera Trapping and a Comprehensive Examination of Survey Data by Village with Duration and Camera Station Counts


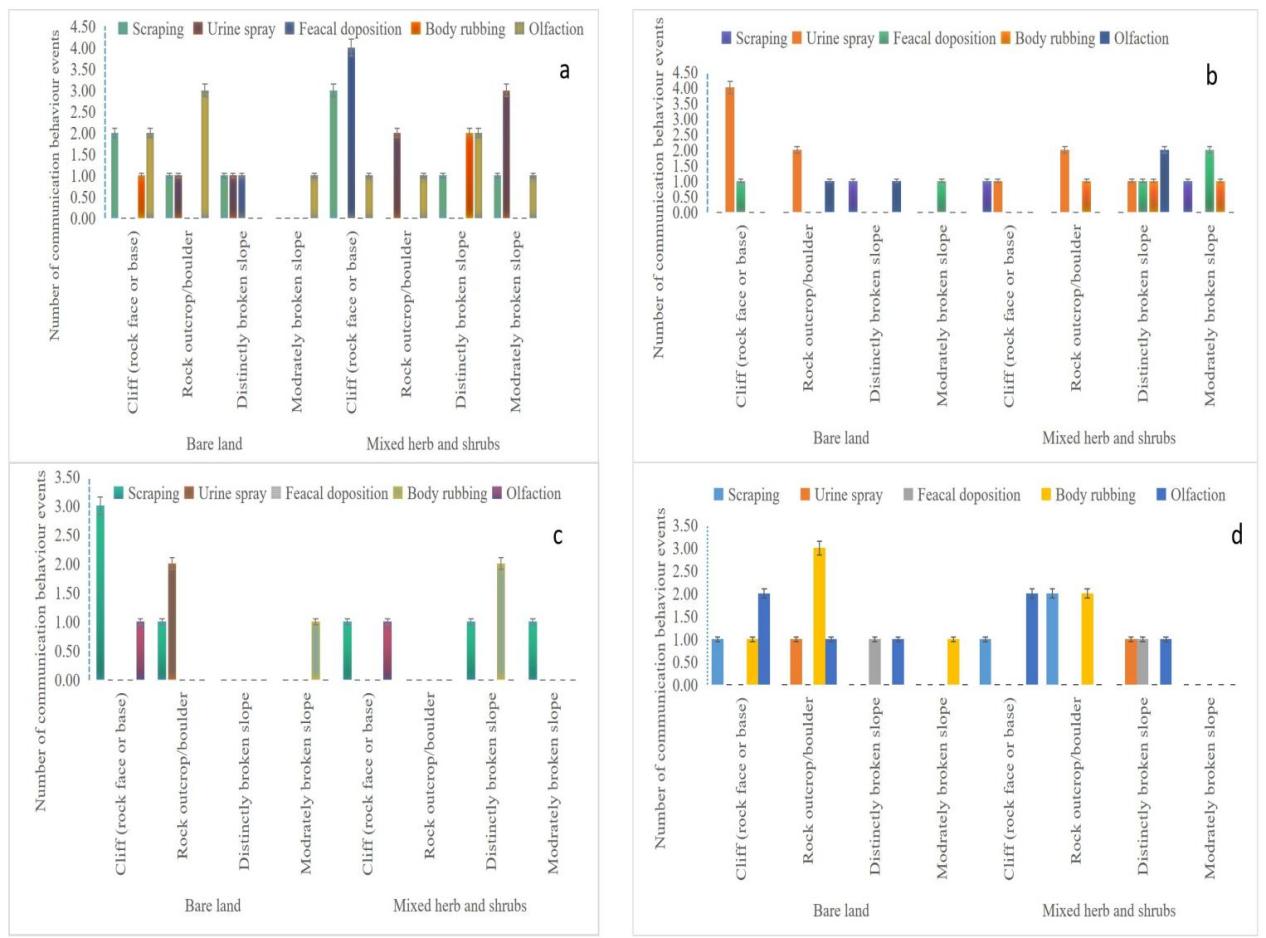


Figure S1: Snow leopard communication behaviour events related to feature marked and terrain type such as cliffs, rock outcrops, distinctly broken slopes, and moderately broken slopes during the absence of snow for (a) nighttime and (b) daytime and in the presence of snow for (c) nighttime and (d) daytime.

Table S2: The study compared snow presence/absence day or night, marking time and communication behaviour of snow leopards in different habitats using the Mann-Whitney Test (U), Z-test. Significance levels were determined based on marking activities during snowy and snow-free conditions.

| Grouping Variable Day vs Night | Type of Marking Behaviour | U | Z | Sig. |
| --- | --- | --- | --- | --- |
| a) Absence of Snow, Night vs Day | Scent Marking | 111.00 | -0.17 | 0.85 |
|  | Body Rubbing | 108.00 | -0.73 | 0.46 |
|  | Investigating | 103.01 | -0.65 | 0.51 |
| b) Presence of Snow Night vs Day | Scent Marking | 529.00 | -1.83 | 0.06 |
|  | Body Rubbing | 647.00 | -0.05 | 0.95 |
|  | Investigating | 611.00 | -1.26 | 0.20 |
| c) Snow Absence vs Presence Day | Scent Marking | 689.00 | -3.35 | 0.00 |
|  | Body Rubbing | 100.00 | -0.33 | 0.73 |
|  | Investigating | 961.50 | -1.03 | 0.30 |
| d) Snow Absence vs Presence Night | Scent Marking | 581.00 | -1.43 | 0.15 |
|  | Body Rubbing | 660.00 | -0.94 | 0.34 |
|  | Investigating | 585.00 | -2.19 | 0.02 |


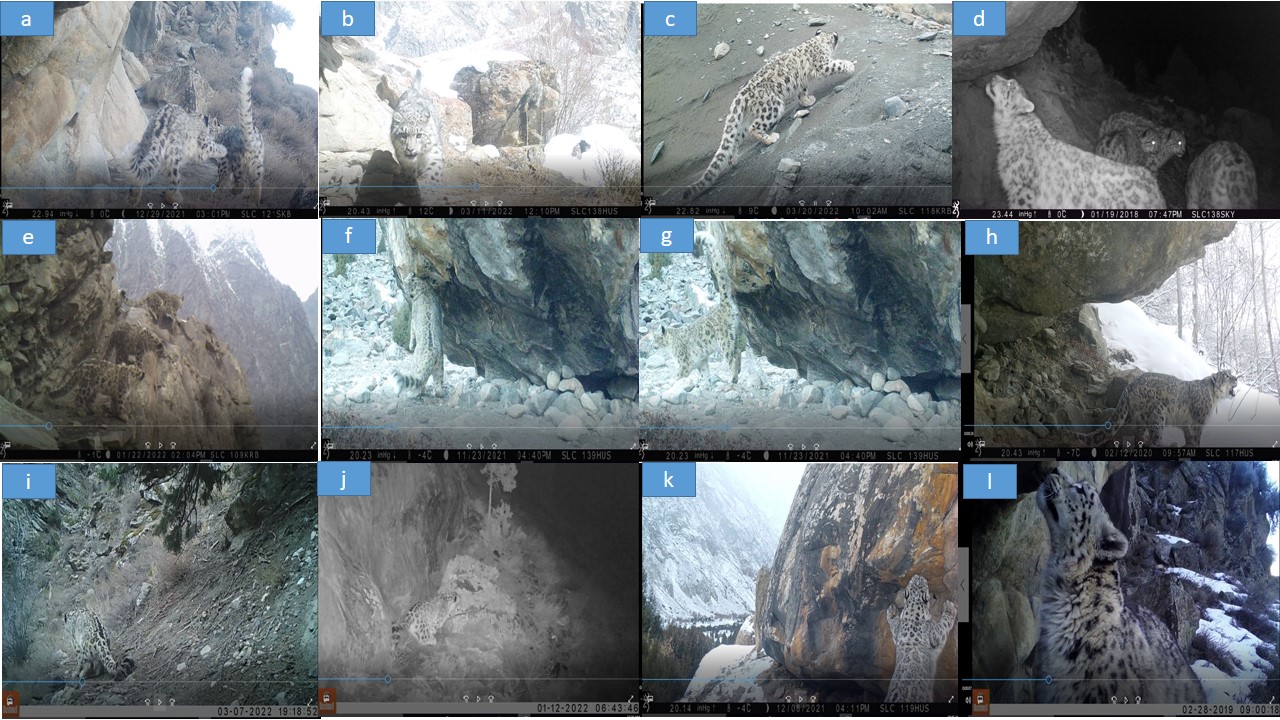


Figure S2. The first photographic evidence showcasing various communication behaviours and the observation of snow leopard cubs with their mother in northern Pakistan has been documented. (a) Three sub-adult cubs were observed in the Skoyo valley. (b) One sub-adult cub was spotted in the Hushe valley. (c) Another sub-adult cub was documented at Trabathang. (d) A female with three cubs was recorded in Rundo. (e) An adult with three cubs was also seen in Rondo. (f) An adult snow leopard exhibited cheek rubbing behaviour in Hushe. (g) Another adult snow leopard displayed urine spraying behaviour in Hushe. (h) Various vocalization behaviours were observed in Hushe. (i) The scat deposition and defecation behaviours of snow leopards were studied. (j) An adult leopard demonstrated scraping behaviours in Rondo. (k) Snow leopards exhibited olfaction or sniffing and claw marking behaviour in Hushe. (l) Sniffing behaviour to observe cues and signals was documented in Rondo.
