# Supplementary material for: Silent Signals in the Snow: Tracking the Spatio‐Temporal Territorial Marking Behavior of Snow Leopards (Panthera uncia) in the Mountainous Region of Baltistan, Pakistan
Source: Ecol Evol. 2024 Dec 11;14(12):e70518. doi: 10.1002/ece3.70518 (PMC11634815; doi:10.1002/ece3.70518)
Supplement: Supplementary file 1 — Data S1. [file ECE3-14-e70518-s001.zip › r script for model.docx]

2=read.csv(file.choose(),header = T)

library(overlap)

library(circular)

timeRad=a2$time*2*pi

head(a2)

tail(a2)

night=timeRad[a2$zone==1&a2$sps=="markhor"]

day=timeRad[a2$zone==2&a2$sps=="ibex"]

par(mfrow=c(2,2))

a3=read.csv(file.choose(),header = T)

library(overlap)

timeRad=a3$time*2*pi

head(a3)

tail(a3)

meso=timeRad[a3$zone==1&a3$sps=="fox"]

lard=timeRad[a3$zone==2&a3$sps=="leopard"]

crt=timeRad[a3$zone==3&a3$sps=="marten"]

densityPlot(day,night,rug=TRUE,xcenter = (a),(Arg = NULL))

overlapPlot(lard,meso,ylab="Temporal activity patterns",ylim=c(0.0, 0.15),xlab="Time of activity", main="a")

legend('topright', c("Leopard","Fox"), lty=c(1, 2), col=c("black", "blue"), bty='n')

legend('topleft',c("Presence of snow"),bty='n')

abline(v=c(6.00, 18.00+00/60), lty=3)

length(meso)

length(lard)

( Dhats <- overlapEst(meso, lard) ) # or just get Dhat1

( Dhat1 <- overlapEst(meso, lard, type="Dhat1") )

# Do 999 smoothed bootstrap values:

LeopardFox<- bootstrap(meso, lard, 999, type="Dhat1")

mean(LeopardFox)

hist(LeopardFox,ylab="Detention frequency of temporal activity patterns",ylim=c(0,300),xlim=c(0.5, 0.9), main="e")

abline(v=Dhat1, col='red', lwd=2)

abline(v=mean(LeopardFox), col='blue', lwd=2, lty=3)

# Get confidence intervals:

bootCI(Dhat1,LeopardFox)['norm0', ]

bootCI(Dhat1, LeopardFox)['basic0', ]

bootCI(Dhat1,LeopardMarten )['norm0', ]

bootCI(Dhat1,LeopardMarten )['basic0', ]

library(MuMIn)

library(lme4)

We used model construction by using glm with binomial error

a=glm(leom~ ddblf +mf+ws+gl+dc+sr+tr,data = leoa, family="binomial")

> summary(a)

> step(a)

This is used for calculated confidence interval with 95% and exponent coefficient

> #OR and 95% CI

> exp(coef(e))

> exp(confint(a))

Calculate AIC for each models

> AICc<-AIC(a)

> loglivehoods calculation for model.

logLik(a)

Delta AICC calculate methods calculated by highest value minus low values

> Da<-AIC(a)-AIC(e)

Calcuted methods for weights of models

> wa<-exp(-Da/2)

> wa

> sumdiccc<-exp(-Da/2)+exp(-Db/2)+exp(-Dc/2)+exp(-Dd/2)+exp(-de/2)

> sumdiccc

> a<-wa/sum(sumdiccc)

|  |
| --- |
